# External control of reactions in microdroplets

**DOI:** 10.1038/srep11837

**Published:** 2015-07-02

**Authors:** Samaneh Mashaghi, Antoine M. van Oijen

**Affiliations:** 1 Zernike Institute for Advanced Materials, Centre for Synthetic Biology, University of Groningen, Nijenborgh 4, 9747 AG Groningen, The Netherlands; 2 School of Chemistry, University of Wollongong, Wollongong, NSW 2522, Australia

## Abstract

Scale reduction of chemical reactions enables novel screening and synthesis approaches that facilitate a highly parallelized and combinatorial exploration of chemical space. Droplet-based microfluidics have evolved as a powerful platform to allow many chemical reactions within small volumes that each can be controlled and manipulated. A significant technical challenge is the ability to change the concentration of reactants inside a droplet. Here we describe a strategy that relies on the use of reactants that are soluble in both oil and water and allow a passive, diffusive exchange of reactants between the oil and aqueous phases to externally control composition of the droplets. We demonstrate the applicability of our approach by externally changing the pH inside microdroplets without the need for physical manipulation or droplet merging.

Reaction control is an important concept in chemistry and chemical engineering with various applications requiring kinetic control of chemical processes or reaction yields^1^,^2^,^3^,^4^,^5^,^6^,^7^. Typical control parameters include temperature, light, pH, reactant concentration and voltage 8,9. The dynamic nature of the control can vary from a simple on/off switch that triggers or stops a reaction at a well-defined time point to a feedback loop in which the control parameter is determined by an observable from the reaction chamber.

Reduction of the scale of a reaction allows multiplexing and has enabled great advances in chemical screening and combinatorial chemistry. Smaller reaction volumes allow precise control of reaction parameters and thus support reliable comparisons of chemical species across chemical libraries by minimizing unwanted fluctuations in reaction parameters such as concentration. An important approach towards the use of small reaction volumes is droplet-based microfluidics, in which discrete aqueous reaction volumes are produced and manipulated in the oil phase^10^. Easy manipulation, transport and sorting have made droplet-based microfluidic systems ideal for many applications in synthetic and analytical chemistry^11^. Another useful approach is the use of printed microdroplet on a solid support as reaction volumes. Printing arrays of microdroplets on a support can be readily achieved in a reproducible manner, supported by advances in the development of noncontact printers^12^,^13^,^14^.

Performing chemistry in droplets promises high controllability, though technical challenges are still remaining. In particular, changes in droplet content are difficult to achieve and typically require merging of multiple droplets, a process that is highly selective and can be used to mix a wide range of materials but one that is hindered by the high interfacial tension between the discrete aqueous and the continuous oil phase^15^. Further, droplet merging comes with an additional challenge of controlling reactant concentrations with changing volumes. As a result, it is challenging to use merging when the pH of a droplet needs to be adjusted independent of its size and concentration of other reagents. This point becomes particularly important in multi-step reactions where the pH of a droplet has to be adjusted multiple times to different values. Here we present an approach that relies on the use of reactants that are soluble in both the aqueous and oil phase as an alternative method to control the concentration of reactants within a droplet. By dynamically changing the concentration of reactant in the oil phase, precise control of the concentration of the same reactant within the droplet can be achieved without the need for droplet merging. The notion of such a biphasic diffusive exchange was previously used for micro-liquid liquid extraction where two immiscible liquids come into contact and exchange solutes^16^. Furthermore, the transport of reagents to dispersed droplets via diffusive transport from the continuous phase has been exploited previously to initiate polymerization reactions and to precipitate inorganic materials^17^. Even though this strategy has been proven useful to introduce reagents from aqueous phase into oil droplets, the literature on introducing reagents from the water phase to oil phase in a quantitative manner is scarce.

We demonstrate this approach by modulating the internal pH of aqueous droplets in oil. To our knowledge, this is the first quantitative method that allows fast control on the acidity of buffered micro-droplets independent of its size. We use either an acid or base that is soluble in both the aqueous and oil phase and show exquisite control of pH inside the microdroplets by dynamically exposing them to oil phases with varying concentrations of acid or base. We demonstrate the applicability of this approach in solutions of emulsion droplets, in static droplets deposited on solid surfaces, and in dynamic droplets generated in and moving through microfluidic structures. Importantly, our method allows the concentrations of micro-droplet based reactants to be maintained during the pH-switching process and can be applied at any point in a multi-step, lab-on-a-chip process.

## Results and Discussion

**Figure 1:**
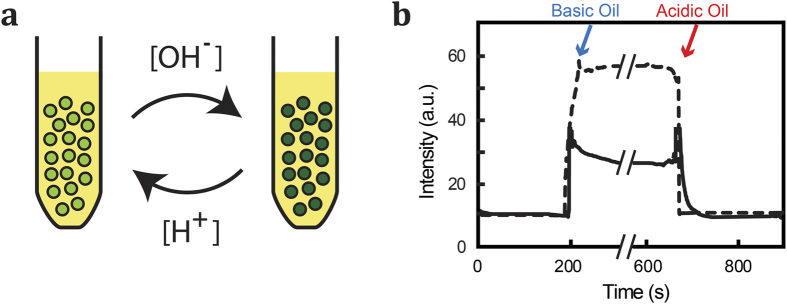
pH control of an emulsion of aqueous droplets in oil. (**a**) The pH of emulsified aqueous droplets is reported by the pH-sensitive fluorescein dye in the aqueous phase (100 μM fluorescein in aqueous buffer:oil in 1:10 V/V ratio). Addition of acetic acid or propyl amine, both compounds soluble in water and oil, to the oil will result in a reduction or increase of the droplet pH, respectively. (**b**) Time trace of the fluorescein emission of the emulsion as propyl amine is introduced to the oil, followed by addition of acetic acid. The increase and decrease of the fluorescein signal corresponds to a rise and fall of the pH inside the droplets. The solid line corresponds to fluorinated oil, the dashed line to silicon oil (the graphs are superimposed from two independent experiments).

First we use an emulsion of aqueous solution in silicone oil to demonstrate that acid and base can be dissolved in the oil phases and transferred efficiently to the aqueous droplets. Based on their amphipathic nature reported solubility in water and organic solvents^18^, we arrived at the use of acetic acid and propyl amine as the acid and base, respectively. We found that both acetic acid and propyl amine display significant solubility in fluorinert oil and silicon oil. However, they display negligible solubility in mineral oil and sunflower oil. By sonication of a mixture (10:1 V/V) of oil and aqueous buffer (pH 7.0; containing100 μM of the pH-sensitive dye fluorescein), we have generated neutral-pH aqueous droplets in oil phase. After preparation of the emulsion by sonication (see Methods section), we add additional oil containing dissolved acetic acid or propyl amine to the emulsions by manual pipetting. Monitoring the fluorescence intensity of the fluorescein revealed a drop in fluorescence intensity upon acid injection, consistent with the fluorescein only emitting fluorescence at a pH above pH 6.7 (Fig. 1)^19^. Upon addition of oil containing the base propyl amine, the fluorescence was recovered.

**Figure 2:**
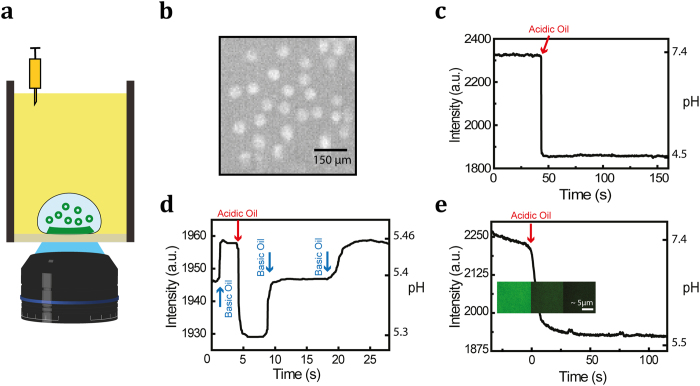
Control of pH in surface-immobilized droplets. (**a**) Schematic of the experimental design. Aqueous droplets inside oil are placed on the top surface of a microscope coverslip. The concentration of base or acid inside the oil phase was altered by addition of propyl amine and acetic acid, respectively. The change of the fluorescein signal inside individual aqueous droplets was measured by total-internal reflection fluorescence microscopy. (**b**) A fluorescence image of micro droplets with a diameter of ~70 μm. The droplets were generated by a T-junction on a microfluidic chip, collected and deposited on a glass surface. (**c**) Fluorescein-containing, surface-immobilized aqueous droplets in silicon oil were exposed to acetic acid, resulting in a rapid decrease in fluorescein intensity. The fluorescence is obtained from a single droplet. (**d**) Fluorescence time trajectory of a single droplet undergoing small pH changes (right axis) upon repeated exposure to acetic acid and propyl amine via the oil phase. pH changes of a few tenths of a unit can easily be achieved and resolved. (**e**) A supported lipid bilayer is established on the water-glass interface inside a droplet and the pH reduced by addition of acetic acid to the oil phase. By coupling the fluorescein pH reporter molecule to the lipid membrane, a change in pH close to the membrane can be measured. Inset shows fluorescence images of a section of the membrane inside a single droplet at−12, 3, and 50 seconds (with the pH change at *t* = 0 s). All experiments are performed in either silicon oil (panels **c** and **e**) or fluorinated oil (panel **d**).

The response time as measured in this experiment corresponds to the dynamics of pH change in a large ensemble of highly heterogeneous droplets. To obtain a better, and more quantitative, understanding of the kinetics of pH change within the droplets, we performed a similar experiment on individual droplets by imaging them while immobilized on a surface (Fig. 2a). By micro pipetting we deposited spatially well-separated micro-droplets containing fluorescein onto the surface of a microscope coverslip covered with oil (Fig. 2b). Figure 2c shows that the pH within a single droplet can be changed on the timescale of seconds. The fluorescence trajectory depicted in Fig. 2d demonstrates large and highly controlled changes in fluorescence intensity of single droplets even when changing the pH by only tenths of a unit.

Micro-droplet printing technology^20^ can be combined with our pH control strategy to generate droplets with identical sizes, with defined positions and with applicability to both solution and surface chemistry. One can divide a large planar surface into many independent reaction zones by printing nano/picoliter-size aqueous droplets on the surface, covered by an oil phase. The surface can be functionalized as desired, being either inert or reactive. Such platform not only allows high-throughput surface chemistry but also allows for monitoring solution reactions using surface-sensitive techniques such as TIRF and SERS. One such application can be found in the field of membrane biotechnology. To visualize and study processes taking place in the membrane, one approach is to place native or model membranes on a solid support^21^. Here, we produce a fluid supported lipid bilayer within a static, surface-supported droplet and show controllability of pH within such system.

We establish a lipid bilayer on a microscope coverslip within a droplet by introducing liposomes into the aqueous phase. The liposomes bind to the surface, merge, and splay out to form a planar lipid bilayer^22^,^23^. The bilayer contains 1% fluorescein-labeled phospholipid that allows us to measure the pH near the membrane. Upon introducing acid to the oil phase, the pH at the surface changes within seconds (Fig. 2e).

**Figure 3:**
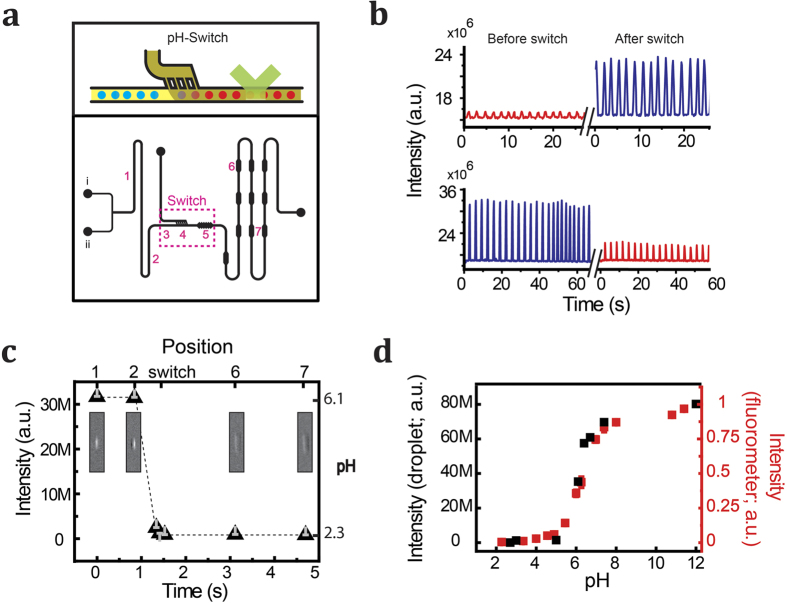
Microfluidic switching of droplet pH. (**a**) Design of microfluidic device. Continuous oil and aqueous phases enter device via inlets (i) and (ii), respectively. Droplets of desired size are generated in a head-on configuration and travel via (1) and (2) to the switch (4) (also schematically depicted in top panel). After combining the initial continuous phase with oil containing acid or base, the droplets pass area (5) where mixing of the two oils takes place (light and dark yellow in top panel). Subsequently, the droplets travel through a serpentine channel containing small reservoirs to temporarily slow the droplets down (6, 7) to prevent blurring during fluorescence imaging. (**b**) Fluorescence time traces acquired at positions before the switch (position (1)) and after the switch (position (6)). The peaks represent individual, fluorescein-containing droplets passing the detection area. The top panel represents a switch from pH 5.5 to pH 6.0, resulting in an increase in the fluorescein signal. The bottom panel represents the converse experiment, decreasing the pH from 6.1 to 5.8. c) Fluorescence of droplets as measured at the various positions indicated in (**a**). The top axis indicates the position on the chip, the bottom axis the relative time of passage. Effective switching takes place on a 100-ms time scale. (**d**) To relate pH after the switch to the acid/base concentration in the second oil phase, the fluorescence intensity of fluorescein-containing droplets with known pH was measured (black) and compared to the fluorescence of droplets after exposing them in the switch to oil containing different concentrations of acid or base. Using this calibration curve, the acid/base concentration needed to reach a target pH in the switch can be accurately determined. Shown in red is the calibration curve measured by the fluorometer.

Next, we set out to demonstrate whether we can control the pH of droplets produced and manipulated within emulsion-based microfluidic structures. A bright-field microscopy image of the structure is given in Figure S1. Our strategy is to bring droplets into contact with an oil phase containing acids/bases, allowing an exchange of acid and base between the oil phase and the droplet to modulate the pH of the aqueous phase (Fig. 3a). Using a microfluidic device, pH-neutral aqueous droplets of desired size and shape are generated in a carrier oil phase (Methods section). Subsequently, the pH of the droplets is changed by having them travel past an on-chip reservoir of oil containing the acid or base (Fig. 3a). Mixing of the ‘neutral’ oil phase with the one containing acid or base, followed by diffusion of the acid or base into the aqueous droplet, will cause a rapid change of droplet pH.

Comparing the fluorescence intensity of fluorescein-containing droplets between points before and after the pH switch shows the efficiency of pH change (Fig. 3b). Measuring the fluorescence intensity of individual droplets at various points along the microfluidic channel provides us information on the rate of pH change while passing the pH switch (Fig. 3c). With a sub-second response time, the pH of the droplet can be switched to a desired value. In these experiments, the distance between two consecutive droplets was nearly 5 times the length of one droplet (*L* = 90 μm in the channel). Therefore, droplet pH remains stable as the droplet travels into subsequent modules in the chip. Calibration of the droplet intensity using solutions with known pH (Fig. 3d) allows us quantitative control of the target pH of the droplets in the microfluidic structure.

Transport processes described in this article are governed by a number of parameters; some being relevant to all platforms while others are platform specific. The former group includes composition of oil, type and amount of surfactant^24^, and temperature^25^. For example, increase of the amount of surfactant has been reported to affect exchange of materials between droplets^24^. Here, we highlight the importance of oil composition in determining the pH of the aqueous phase (Figure S2). Depending on the specific applications and design of the platforms, it is necessary to adjust these parameters on a case-by-case basis to achieve the desired functionality.

**Figure 4:**
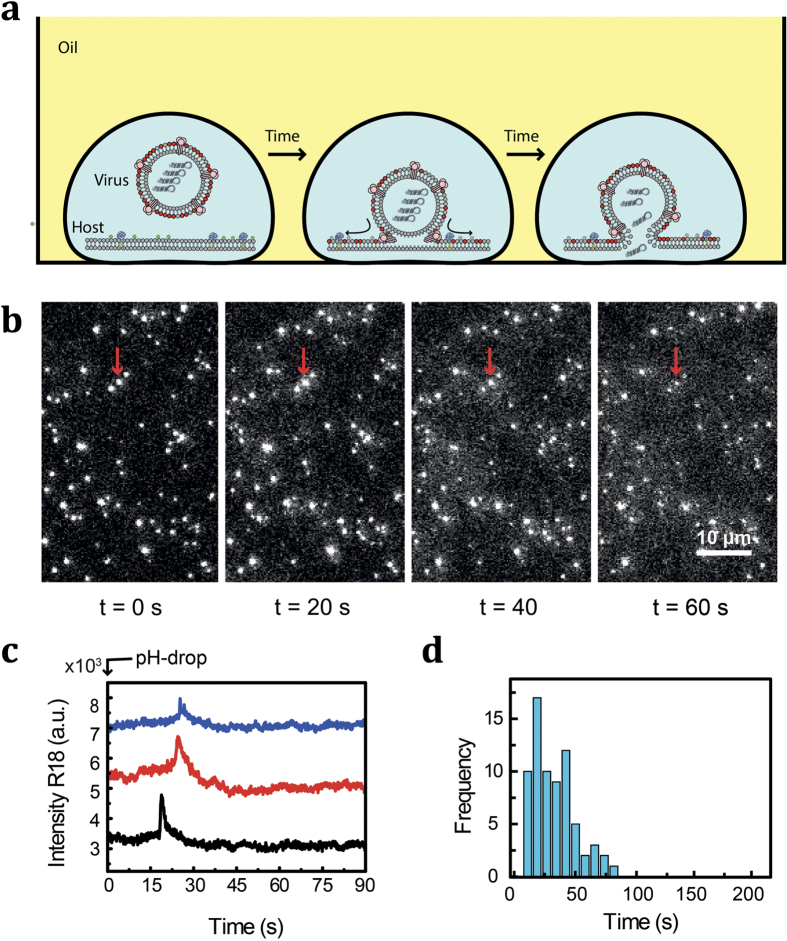
Single-particle monitoring of viral fusion events within microdroplets. (**a**) Schematic illustration of viral fusion assay. (**b**) Fluorescence image sequence of viruses bound to membrane at different time points: upon acidification (*t* = 0 s), and during fusion (*t* = 20, 40, 60 s). Acidification is signified by reduction of membrane fluorescence intensity (with the pH-sensitive fluorescein as a membrane marker). The fusion process is visible as the sharp R18 fluorescence spots temporarily gaining intensity and becoming diffuse. The scale bar is 10 μm. (**c**) Representative fluorescence traces of individual viral particles. Individual traces are offset in the vertical dimension for clarity. (**d**) The histogram of time delays measured at pH 5.0.

Finally, we show that our approach can be used to perform complex pH-triggered reactions in micro-droplets. Here we chose to study fusion of membrane-enveloped viruses with a supported-lipid target bilayer in static micro-droplets that are immobilized on a solid support. Gaining entry of viral particles into cells represents the first step in the infection pathway. For membrane-enveloped viruses, a key step in the entry process is the fusion of the viral lipid bilayer with the membrane of the host cell. For a large number of membrane-enveloped viruses (such as influenza and dengue), the viral particles are taken up into cell via endosomal uptake pathways and the fusion process is triggered by the low-pH milieu in the late endosome. For the influenza virus, the surface protein hemagglutinin (HA) mediates both anchoring the virus to a cellular target and fusion of the virus membrane with the target membrane. In this process, large conformational changes in HA are triggered by low pH and bring the two membranes in such close proximity that the large activation barrier to fusion is overcome (Fig. 4a). Here we first printed microdroplets containing labeled viral particles and liposomes (see Methods section) onto a glass slide covered by oil. Figure 4b provides a typical image of viral particles and the change introduced by their fusion to the supported planar membrane after subjecting the oil phase to acidification. As can be seen in Fig. 4c, the R18 fluorescence intensity increases with a time delay after acidification. We attribute the increase in fluorescence signal of R18 to dequenching, a process that occurs when hemifusion merges the two lipid bilayers and allows escape of the dye into the planar bilayer. Figure 4d shows a histogram of times elapsed after the pH drop for single particles to fuse. The shape and characteristic times correspond with the kinetic data obtained from previously reported single-particle fusion experiments^26^. Our ability to perform this assay in microdroplets potentially enables the screening of a large number of conditions by microprinting large arrays of microdroplets on a solid surface and triggering and observing the fusion kinetics in each droplet.

## Conclusions

We have described here an alternative method towards manipulating the chemical content of emulsion droplets, an approach that we believe will find many useful applications in droplet-based microfluidics. We demonstrated its experimental feasibility by exerting fast and quantitative control over the pH of emulsion droplets. The technical simplicity of our method has advantages compared to active strategies such as electrolysis. Further, the change in the pH does not lead to change in the droplet volume and thus our method does not suffer from the side effects of droplet merging strategies. Central to our microfluidic approach are the solubility, diffusivity and partitioning of the acid and base. Only when a compound is used that is both water and oil soluble, molecular transfer to the droplet will occur. Solubility, partitioning and the volumetric flow rates of the compound in the two oil phases determine the pH range that can be obtained. Additionally, the dynamic response will be affected by the speed of droplets and the geometric properties of the microfluidic switch.

An important engineering principle in microfluidics is modularity. A microfluidic chip is often composed of multiple modules in which different processes such as chemical reactions, change of physical parameters, analysis, sampling, and separation take place. Our switching approach is compatible with different microfluidic designs and can be extended to not only change the pH of droplets but also the chemical content of the droplets in general. Oil-water soluble redox reagents, chelating agents and catalysts can be used to allow fast and passive switching of reaction conditions inside droplets and further extend the applicability of microfluidic approaches to chemical screening and synthesis.

## Methods

Two oils were used in this study: fluorinert-based oil and silicon oil. The fluorinert-based oil was prepared by mixing 3 M Novec^TM^ 7500 (Sphere Fluidics) and Fluorinert^TM^ inert FC-40 (Pico-Surf^TM^ series; Sphere Fluidics) from Dolomite with the ratio of 50:1 V/V. The commercially bought FluorinertTM inert FC-40, contains 5% fluorosurfactants that was commercially synthesized by coupling oligomeric perfluorinated polyethers (PFPE) with polyethyleneglycol (PEG). The surfactant is known to adsorb to the interface forming an interfacial surfactant layer, which stabilizes the emulsion and prevents the adsorption of biomolecules to the interface^27^. The silicon oil was used as obtained from Hampton Research. Oil with various concentrations of acid or base were made by mixing of acetic acid (glacial) 100% (Sigma-Aldrich) or propyl amine (Sigma-Aldrich) with oil, respectively.

### Bulk emulsions

Emulsion of aqueous droplets in oil was prepared by sonication of 100 μM fluorescein (Invitrogen) in HNE buffer (5.0 mM Hepes, 140 mM NaCl, 0.2 mM EDTA, pH 7.4) mixed with the oil at 1:10 V/V for 3 hr. The fluorescence intensity of the fluorescein-containing droplets was measured over time using a fluorometer while stirring (at 800 rpm). During the measurement, the pH of the system was adjusted to the desired pH by injection of acidic and basic oil into the oil phase of the emulsion using a syringe. In order to obtain a pH calibration for the bulk emulsion experiments, we measured the intensity of the fluorescein emulsions at various known pH values.

### Surface-immobilized droplets

Experiments were performed in 8-well-chambered glass slide. The glass surface was cleaned by subsequently rinsing it with acetone, ethanol (Sigma-Aldrich) and Milli-Q water, followed by an etching of the surface by oxygen plasma. The surface of the glass was silanized by incubating the coverslips in a solution of 2% (v/v) 3-aminopropyltriethoxysilane in acetone for 5 minutes. The reaction was then quenched by rinsing the coverslips with Milli-Q water. The coverslips were dried by incubating them in 110 °C for 45 minutes.

In all experiments, the total reaction volume was 100 μl (droplets plus oil). Droplets either contained aqueous solution of fluorescein (2 μg/ml) from (Sigma-Aldrich) for pH change experiments or liposomes for the membrane experiments. The liposomes were prepared by mixing the lipids in chloroform with a molar ratio of 80:19:1 percent of DOPC: Cholesterol: fluorescein-PE (Avanti Polar Lipids, Inc.). The chloroform was removed from the lipids by first evaporating the chloroform under an argon stream, followed by desiccation for 2 hours. Subsequently, the lipids were suspended in HNE buffer to 50 mg/ml. The lipids were then subjected to 10 freeze/thaw cycles and subsequently extruded using 0.2 μm pore size polycarbonate membranes.

For the pH-change experiments, the droplets containing 1 nl of fluorescein (2 μg/ml in HNE) were prepared using a T-junction microfluidic chip. Subsequently, the droplets were transferred to the bottom of a chambered slide containing oil. For the membrane experiment, the droplets containing liposomes were printed on the glass surface by micro-pipetting, followed by filling of the chamber with oil to allow wetting of the surface by the droplet. The inverted fluorescence microscope setup used to visualize the fluorescence from the surface-immobilized droplets was equipped with an electron-multiplying CCD camera (Hamamatsu Photonics K.K., Image-EM model C9100-13). A 60X/1.49NA objective (Olympus) was used for the pH change experiments and for TIRF detection of the membrane.

Viral fusion experiments were performed in arrays of micro-droplets deposited manually on a glass support and covered with a layer of silicon oil. The droplets contained viral particles and a planar lipid bilayer generated from fluorescein-labeled liposomes mimicking the host-cell membrane in a HEPES based buffer (HNE; pH 7.4). The fluorescein is only fluorescent above its protonation pKa of 6.8 and acts as a pH indicator. The lipid bilayer of influenza viral particles (X31) was stained with the lipophilic dye rhodamine B (R18). The fusion reaction was triggered by lowering the pH of the medium to 5.0. A home-built TIRF microscope with 60X objective (NA = 1.49) and 531-nm (for the R18) and 488-nm (for the fluorescein) lasers were used for imaging. Images were taken by an EM-CCD camera (Hamamatsu Photonics) with a Dual-View splitter and the microscope was controlled by MetaVue software. The acquired data were then processed to extract kinetic information with ImageJ and home-written Matlab script.

### Emulsion microfluidics

The microfluidic device was made by the standard soft-lithography method of curing an elastomer on a silicon wafer patterned with photoresist. Next, the openings for the inlets and outlets were punched (0.75 mm). The resulting slab was bonded to a thin #1 cover glass (VWR) to close the bottom of the channel. Poly(dimethylsiloxane) (PDMS; Dow Corning) was used to produce the elastomer slabs and SU-8-100 photoresists was used to create channels of 100-μm depth.

The microfluidic design incorporated three inlets, one for injection of the aqueous solution containing the biomaterials, one for injection of the neutral oil and finally, one inlet for the acidic/basic oil, *i.e*. the inlet for the pH-switch. The inlets were connected to a pressure-based microfluidic control system and flow meter (Fluigent) for precise flow control in the channels. The flow rates for the aqueous phase and oil phase were 2.0 μl/min and 3.0 μl/min, respectively. The pressure at the switch inlet was 5.0mbar. We applied a withdrawing pressure corresponding to 5 μl/min to stabilize the microfluidic platform.

The microfluidic design included a droplet generator, the pH-switch module and a number of detection cavities. The microdroplets are formed at the generator and travel to the switch where their pH is adjusted to a desired pH. An operator can externally control the desired pH by changing the concentration of acid (acetic acid) or base (propyl amine) in the oil phase of the switch. Figure S3 show the droplet pH dependency on the content of the switch as described by volume fractions of acid and oil.

Several aqueous solutions of fluorescein (2 μg/ml) in oil (with known different pH values) were prepared and used as the dispersed phase to generate droplets. To measure the calibration curves, we filled the switch with the same oil as of the main channel. As such, the acidity of the droplets (and thus the corresponding fluorescence intensity) traveling through the microfluidic channel remains unchanged. The calibration curve was generated by plotting the intensity as a function of pH.

A wide-field fluorescence microscope was used as the detection system to visualize the droplet fluorescence in the microfluidic structure. A 2x/0.08 NA objective, microscope filter cube (Chroma Ltd. filters zet488m and zt488rpc), AVT Prosilica GX6600 Camera and 488-nm laser (Coherent DPSS) were used.

After collection of the images, we performed the following analysis on the image stacks: First we selected an area in the droplet path at the desired location, large enough to fit maximally one droplet at a time. Next, the integrated intensity inside the selected area was measured for each frame of the stack. The histogram of the integrated intensities collected over time shows two distinct peaks corresponding to the presence and absence of the droplet. The distance between the two peak centers was taken as the fluorescence intensity of the droplet content.

## Additional Information

**How to cite this article**: Mashaghi, S. and van Oijen, A. M. External control of reactions in microdroplets. *Sci. Rep.* **5**, 11837; doi: 10.1038/srep11837 (2015).

## Supplementary Material

Supplementary Movie

Supplementary Information
