# Supplementary material for: External control of reactions in microdroplets
Source: Sci Rep. 2015 Jul 2;5:11837. doi: 10.1038/srep11837 (PMC4488745; doi:10.1038/srep11837)
Supplement: Supplementary Information [file srep11837-s2.pdf]

Supporting Information

External control of reactions in microdroplets

Samaneh Mashaghi[a] and Antoine M. van Oijen[a,b]

[a] *Zernike Institute for Advanced Materials, Centre for Synthetic Biology, University of Groningen, Nijenborgh 4, 9747 AG Groningen, The Netherlands*
[b]*School of Chemistry, University of Wollongong, Wollongong, NSW 2522, Australia*

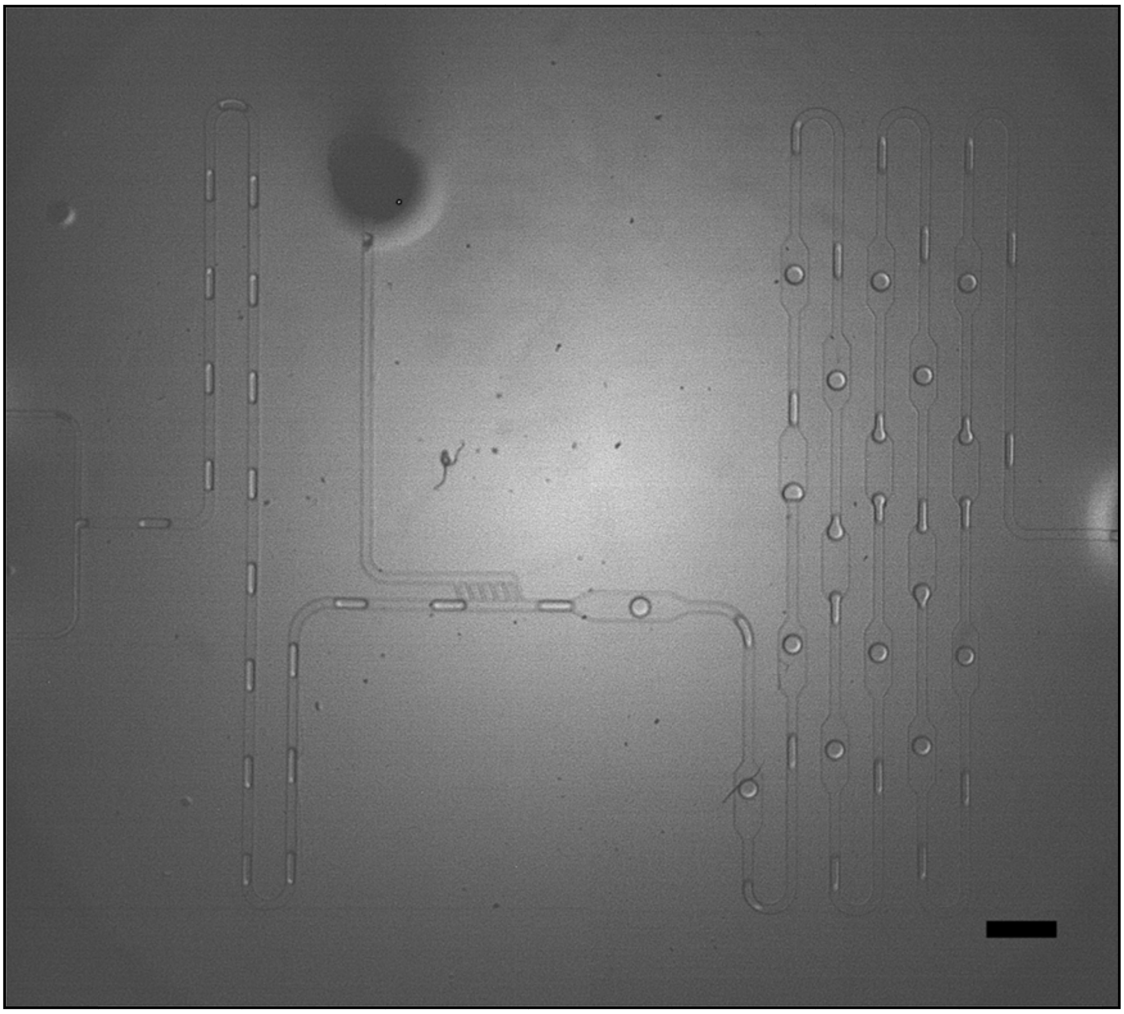

Figure S1: A bright-field image of the microfluidic device. The width of the main channel is 90 µm and the scale bar is 500 µm. A movie of the droplets traveling through the chip can be seen in SMOV1.

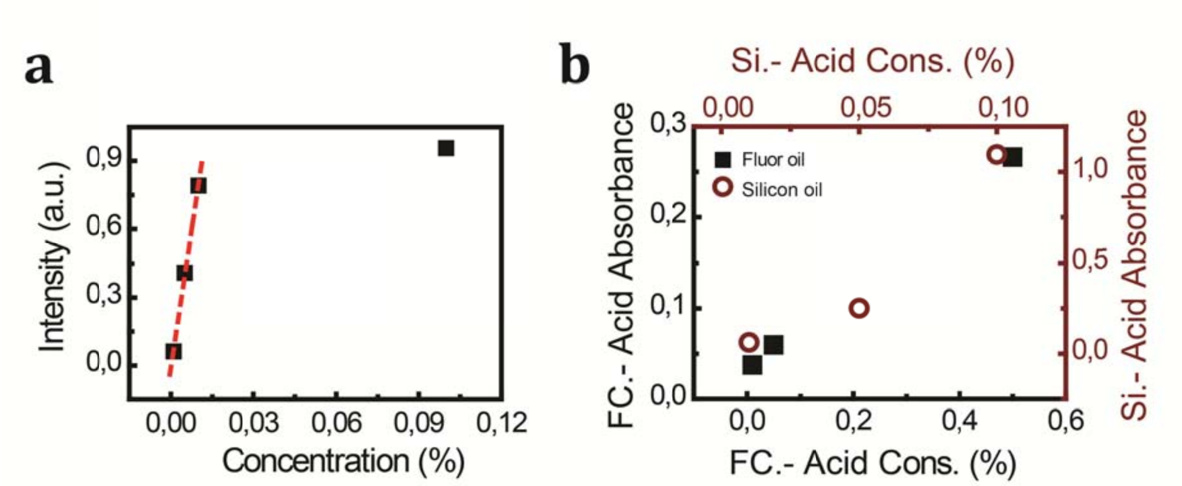

Figure S2: Partitioning of acetic acid between oil and water phases. (a) UV/Vis absorbance of aqueous solutions of acetic acid at λ = 200 nm. By fitting the curve at the regime of low concentrations, we find the following relation between absorbance (A) and concentration (C): A = (80.9 ± 2.8) × C. (b) absorbance at λ = 200 nm of aqueous fraction in a system of equal volumes of water and oil. Two systems are compared: water/fluorinert oil and water/silicone oil.

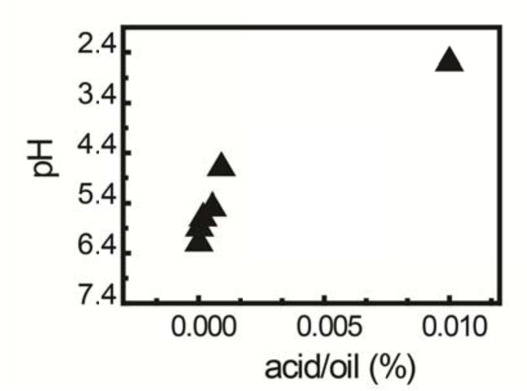

Figure S3: Droplet titration curve depicting the droplet pH after switching as a function of the acid concentration in the switching oil. The change in pH of the droplet content depends on the acid fraction of the oil phase. The aqueous droplet is only weakly buffered by a low concentration of HEPES (5.0 mM, initial, pre-switch pH 7.4).
